# Can intestinal microbiota be associated with non-intestinal cancers?

**DOI:** 10.1038/s41598-017-11644-9

**Published:** 2017-10-05

**Authors:** Camille Jacqueline, Lionel Brazier, Dominique Faugère, François Renaud, Frédéric Thomas, Benjamin Roche

**Affiliations:** 1 CREEC, 911 Avenue Agropolis, BP 64501, 34394 Montpellier Cedex 5, France; 2 MIVEGEC, UMR IRD/CNRS/UM 5290, 911 Avenue Agropolis, BP 64501, 34394 Montpellier Cedex 5, France; 3 International Center for Mathematical and Computational Modeling of Complex Systems (UMI IRD/UPMC UMMISCO), 32 Avenue Henri Varagnat, 93143 Bondy Cedex, France

## Abstract

While the role of intestinal microbiota is increasingly recognized in the etiology of digestive cancers, its effects on the development of cancer in other parts of the body have been little studied. Through new-generation sequencing, we aimed to identify an association between the structure of intestinal microbiota and the presence of eye disc tumor in Drosophila larvae. First, we observed a parental effect on the diversity and structure of bacterial communities. Second, we identified a bacterial signature (at the family level) of cancer: cancerous larvae host a significantly lower relative abundance of Bacillaceae than individuals that did not develop the tumor. Thus, for the first time, we showed that a non-digestive cancer, *i.e.*, in the brain, could be associated with an altered composition of the gut microbial community. Finally, we discuss the potential implications of the immune system in the gut–brain axis concept to explain the long-distant effect of intestinal microbiota on brain tumors. We also highlight the potential of our results in a therapeutic perspective for brain cancer that could be generalized for other cancers.

## Introduction

All human body surfaces and cavities are inhabited by complex communities of micro-organisms (*e.g*., viruses, bacteria) whose composition is influenced by host genetics, feeding habits, life style, and microbial exposure^1,2^. Recent evidence suggests that many human diseases are attributable not only to pathogens circulating in host populations, but also to global changes in our microbiota^3^. Our microbiome contains a metagenome that exceeds our own genome size and interacts with key functions in the homeostasis of our body, resulting in a healthy state. In particular, the microbiome is involved in the modulation of important components of the organism including inflammation and metabolism^4^, which are also considered as two hallmarks of cancer^5^. This is why the intricate interactions of the human microbiome with cancer development have been increasingly investigated, giving rise to the concept of the “oncobiome”^6^. Even though this concept has been criticized because studies have demonstrated associations rather than causations^7^, several investigations in germ-free animals have highlighted the impact of the global microbiota on cancer development in various organs (reviewed by^8^). Because microbial mass is represented at 99% by the bacterial species within the gastrointestinal tract, studies have focused on the role of intestinal microbiota in carcinogenesis.

Changes in intestinal microbiota have been associated with diseases at local (*e.g.*, inflammatory bowel disease^9^) and long-distant (*e.g.*, multiple sclerosis^10^) scales. In the context of cancer, most studies have looked at interactions between intestinal microbiota and digestive cancers. One pro-tumoral role of microbiota is linked to inflammation^11^. For instance, a disruption of the mucosal–epithelial barrier induces pro-inflammatory cytokine secretion, which in turn induces tumor growth in a colon adenoma mice model^12^. In addition, microbiota has been shown to drive colon carcinogenesis through other mechanisms, such as the alteration of retinoic acid metabolism^13^. Microbiota can play a fundamental role in the elaboration of an efficient immune system^14^ and could also promote anti-tumoral surveillance to prevent cancer or at least limit tumor growth. In fact, microbial products shape T cell repertories^15^ and could regulate anti-tumor responses through the priming of cross-reactive T cells specific to both bacterial and tumoral antigens^16^.

Very few studies have explored the link between intestinal microbiota alterations and non-digestive cancers. The pro-tumoral effect of intestinal microbiota has been reported in a liver cancer mice model^17^ whereas reduced bacterial load was positively correlated with breast tumor^18^. Nevertheless, for several reasons (*e.g.*, neural, endocrine, or immune afferents^19^), it is expected that interactions could exist between intestinal microbiota and malignancies located in different parts of the body (*i.e.*, long-distant interactions). For example, the concept of the gut–brain axis describes the complex and bidirectional communication system between brain and gut^20^. The disturbance of this system has been implicated in a wide range of health disorders (*e.g.*, gastrointestinal inflammation, eating disorders)^21^. In the context of cancer, this gut–brain crosstalk has been little investigated even though it seems worth exploring, since immunity—through the production of cytokines—is involved in this communication^22^. Therefore, one could expect that immune responses directed towards cancer cells in the brain could affect the microbial community in the gut, or that alteration in microbiota structure could result in cancer proliferation.

In this study, we explored the possibility of a long-distant crosstalk between a tumor in the brain and intestinal microbiota. We characterized the diversity and structure of intestinal microbiota in Drosophila larvae with a tumor in the eye-antennal disc using new generation sequencing (NGS) of polymerase chain reaction (PCR)-generated amplicons from the 16S rRNA gene. Using microbial community diversity analyses, we sought the presence of a specific signature at the family level that could be linked with the cancerous status. Finally, we discuss the potential implication of the immune system to explain the long-distance effect of intestinal microbiota on brain tumor and highlight the potential of our results in a therapeutic perspective.

## Materials and Methods

### Tumor model and Drosophila stocks

**Figure 1:**
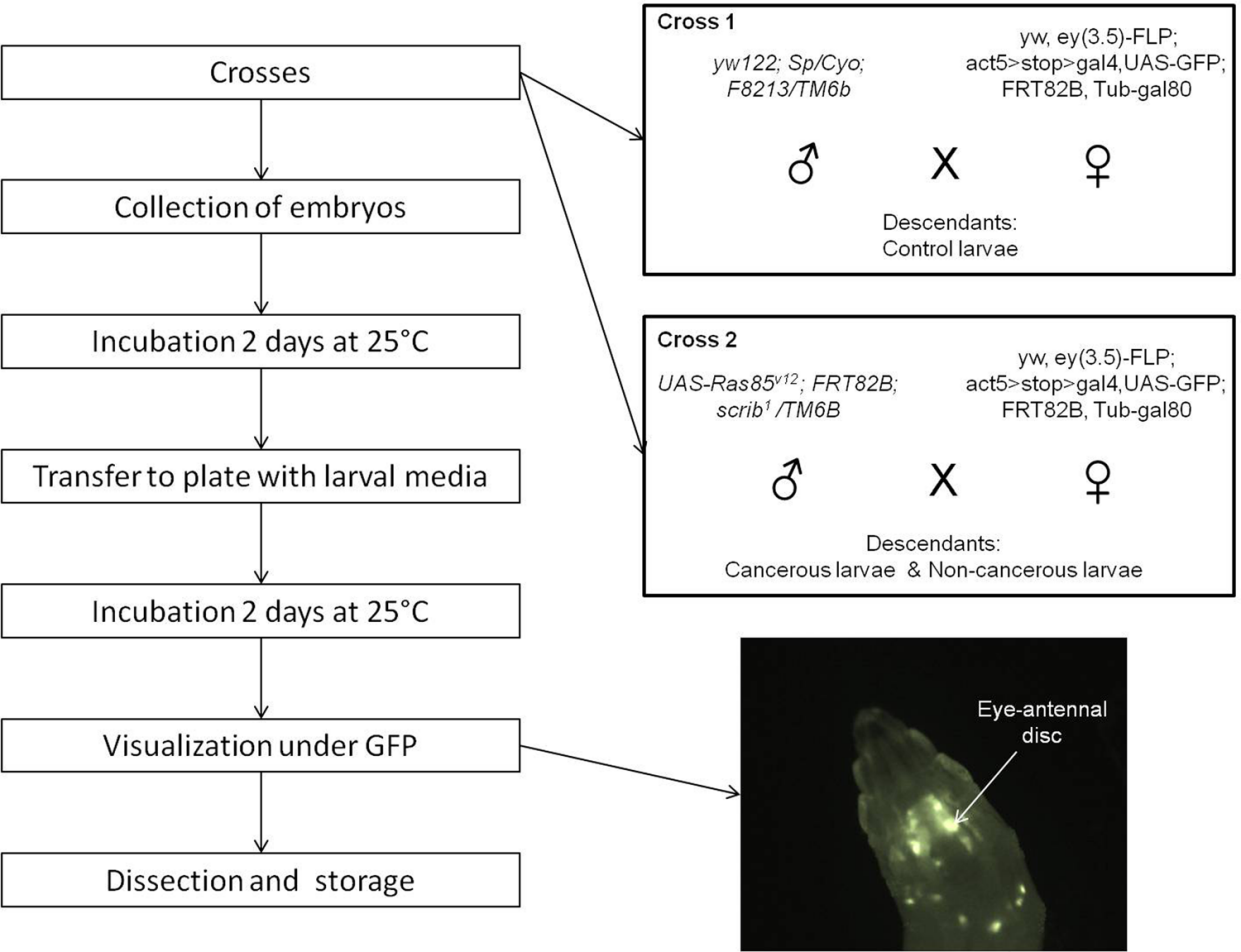
Schematic representation of the experimental design. The two boxes describe the crosses generated to obtain control larvae (cross 1) and cancerous/non-cancerous larvae (cross 2). The picture shows an eye-antennal tumor *in situ* under GFP.

The genetically modified *Drosophila melanogaster* flies used in this study were engineered to develop a tumor of the eye disc, as previously described^23^. The genetic scheme uses *eyeless* promoter-driven FLP recombinase expression (eyFLP) to introduce multiple genetic alterations in GFP-labeled cells, specifically in the developing larval eye-antennal imaginal discs as well as the optic lobes of the brain, but not in other adjacent tissues. Clones are mutant for the cell polarity regulator *scribbled* (scrib), which is a loss-of-function allele. Scrib mutants normally die; however, in combination with oncogenic UAS-Ras85^v12^ (gain-of-function transgene), cell death is prevented and tumor overgrowth occurs. Male yw122; Sp/Cyo; F8213/TM6 flies were crossed with yw, ey(3.5)-FLP; act5 > stop > gal4,UAS-GFP; FRT82B, Tub-gal80 females to generate healthy clones (cross 1). Male UAS-Ras85^v12^; FRT82B; scrib^1^/TM6B flies were crossed with the same females described above to generate RAS/scrib GFP-labeled clones (cross 2) (Fig. 1). Female flies were isolated before emergence at the pupal stage and fertilized by two-day-old males. All Drosophila stocks and crosses were maintained on standard fly media.

### Experimental protocol

The crosses were allowed to lay eggs on sugar-agar plates for less than 24 h, and collected embryos were incubated for two days at 24 °C. Microbiota-free larvae from crosses 1 and 2 were also generated by dechorionation of embryos with 50% bleach. This control allows us to determine contamination that occurs during dissection due to bacteria present on the cuticle or in the hemolymph. From the two crosses, we selected larvae at the late 2^nd^ or early 3^rd^ instar stage based on the lack of the dominant Tubby phenotype carried on the *TM6b* balancer chromosome. After selection, seven larvae were transferred into each well of a 96-well plate filled with yeast-sugar-agar media; wells were then sealed by plugs.

After two more days of incubation at 24 °C, we performed tumor visualization of 3^rd^ instar larvae using a dissecting microscope under GFP fluorescence (Zeiss A Lovert 200 M, 2.5X). The GFP-labeled tumor cells were easily observable in living larvae through the transparent cuticle (Fig. 1). Cross 1 descendants did not show an over-proliferation of eye-disc cells, but half of the cells are GFP-labeled (these control larvae are henceforth referred to NC). Our model has shown that only 20% of the non-Tubby cross 2 descendants harbor the tumor. Thus, we distinguished cross 2 larvae that did not express the tumor (non-cancerous larvae; henceforth referred to as G) from those showing the GFP-labeled tumor (cancerous larvae; henceforth C).

After determining the cancerous status, we obtained 30 larvae for each group (cancerous, non-cancerous, and control) and three control microbiota-free larvae of each cross. Before dissection, each 3^rd^ instar larva was washed in three successive bathes: bleach, 70% alcohol, and sterile water. The gut (from proventriculus to rectum, excluding Malpighian tubules) was then dissected with bleach-sterilized forceps in sterile PBS. The dissected intestines were transferred to Eppendorf tubes for DNA extraction and kept at −20 °C. A control for the dissection was made using a drop of PBS solution treated as for dissections, but without insect materials, to ensure the absence of bacterial contaminants.

### DNA extraction and PCR reaction

Total genomic DNA was extracted from isolated larva guts following the manufacturer’s protocol (QIAGEN DNeasy® Blood and Tissue Kit). All DNA extractions were resuspended in 50 µL of sterile water and stored at −20 °C. Extraction controls were processed in parallel during the DNA extraction procedure to monitor reagent contamination^24^.

The V3 region of the partial 16S rRNA gene (180 pb) was amplified from extracted DNA using the broad-range bacterial-specific primers Probio_Uni and Probio_Rev (Table S1 in supplementary material)^25^. These primers amplify more than 94% of the bacterial 16S rRNA coding sequence. Primers were modified to include a 10-mer barcode tag (forward primer) and adapter sequences for the Ion Torrent PGM sequencer. Each sample, including controls, was amplified by PCR once and identified with a specific barcode. The PCR was performed according to manufacturer’s protocol for Q5® DNA Polymerase (New England Biolabs). PCRs were conducted in 10 µM of each primer and 10 ng of DNA sample in 25 µL final volume. The PCR conditions used were 94 °C for 5 min followed by 22 cycles of 94 °C for 30 s, 50 °C for 15 s, and 72 °C for 15 s, with a final extension step at 72 °C for 8 min. For each PCR plate, we added a negative control tube containing sterile water.

### Ion Torrent PGM 16S metagenomic sequencing

The DNA library constructions derived from PCR were purified twice by magnetic separation using Agencourt AMPure XP DNA purification beads (Beckman Coulter Genomics). The elimination of free primers and new concentrations of libraries were then verified with electrophoresis on an Agilent 2100 Bioanalyser (Agilent Technology). All amplicons were diluted to 26 pM—except for controls (dissection [1 sample], extraction [3 samples], and PCR [2 samples]), which were used pure—and pooled to equalize concentrations for emulsion PCR with the Ion One Touch 400 Template kit v2 (Life Technologies) according to the manufacturer’s instructions. Sequencing of the amplicon libraries was carried using the Ion PGM™ Hi-Q™ Sequencing kit with 48 barcoded amplicon libraries pooled on Ion 318™ chips.

### Sequence filtering

After sequencing, the flowgram files for each sample of each chip were downloaded from the Torrent Server and were then processed using Mothur v1.35.1^26^. The standard operating procedure (SOP) for 454 pyrosequencing was adapted to the Ion Torrent technology^27^. Sequences were filtered to keep sequences with a) homopolymer of a maximum of 10 bases, b) a quality window average ≥30, c) a quality window size of 50 bases, d) one allowed mismatch in primers, and e) one allowed mismatch in barcode. All the *fasta* and *qual* files were concatenated into one file respectively from which were performed: a) alignment of sequences using Silva SSU Reference alignment v102^28^, b) removal of error and chimeric sequences with the uchime program, c) assignment of sequences using the Wang method^29^ and with a bootstrap value superior to 80%, d) clustering into operational taxonomic units (OTUs) with a 97% similarity compared to the reference database, as commonly used^30^, e) filtering and removal of OTUs represented by a single sequence, and f) the consensus taxonomic classification for each OTU.

### Statistical analyses

The shared and taxonomy files generated by Mothur were imported into R with the “phyloseq” package^31^. We considered taxonomic classification up to the family rank because information at species level was too sparse and would introduce bias in diversity analyses due to the large number of unclassified species. In fact, 16 S approaches offer limited taxonomical resolution, particularly when the fragment considered is short, and it has been observed that none of the 16S pipelines performed satisfactorily at the species level^32,33^. Except if it is specified, our analyses, that aimed to generate descriptors of global diversity, were conducted using the total number of classified OTUs. Rarefaction curves for each group of larvae were plotted using observed OTUs and the Shannon index of species richness (the “iNEXT” and “DiversitySampler” packages;^34,35^). In addition, to investigate how our sequencing efforts related to the real bacterial population, we calculated Good’s coverage estimator using the data generated by Mothur (*i.e.*, the number of OTUs represented by a single sequence removed at step e)^36^.

We then characterized the microbiota structure for each group of larvae. To standardize by sampling effort, alpha diversity and Simpson’s evenness index were calculated on a subsample (with replacement) of our libraries using the minimum number of reads found in our samples. We repeated this 100 times and averaged the diversity estimates from each trial. This method is also recommended to control for sequencing errors that could remains after filtering. Principal coordinate analysis (PCoA) (with the *ordination* function in R, using the bray distance method in the “phyloseq” package) and principal component analysis (PCA) (with the *prcomp* function in R) were also conducted on all OTUs to assess structure differences between groups. Finally, after having quantified the impact of parental effects on the descendants’ microbiota, we focused on the effect of the cancerous status on microbiota diversity by comparing cancerous *vs* non-cancerous groups. Redundancy analysis is a common ordination technique in community ecology that allows an unweighted linear regression on constrained variables. RDA was performed using cancerous status (presence or absence of tumor) as the constrained variable and relative abundance of OTUs as the response variable using the *rda* function of the “vegan” package. For this analysis, we focused on the most abundant families, with a relative abundance superior to 0.02% (*i.e.*, 48 OTUs), in order to avoid attributing differences to OTUs that could represent sequencing errors. The significance of the constrained ordination model was assessed by the Monte Carlo Permutation Test. For families identified during this step, we evaluated whether their relative abundances were significantly different between the two groups through non-parametric Mann-Whitney tests and applied Šidák correction for multiple comparisons^37^. All analyses were conducted in R version 3.3.2^38^.

## Results

### Data summary

**Figure 2:**
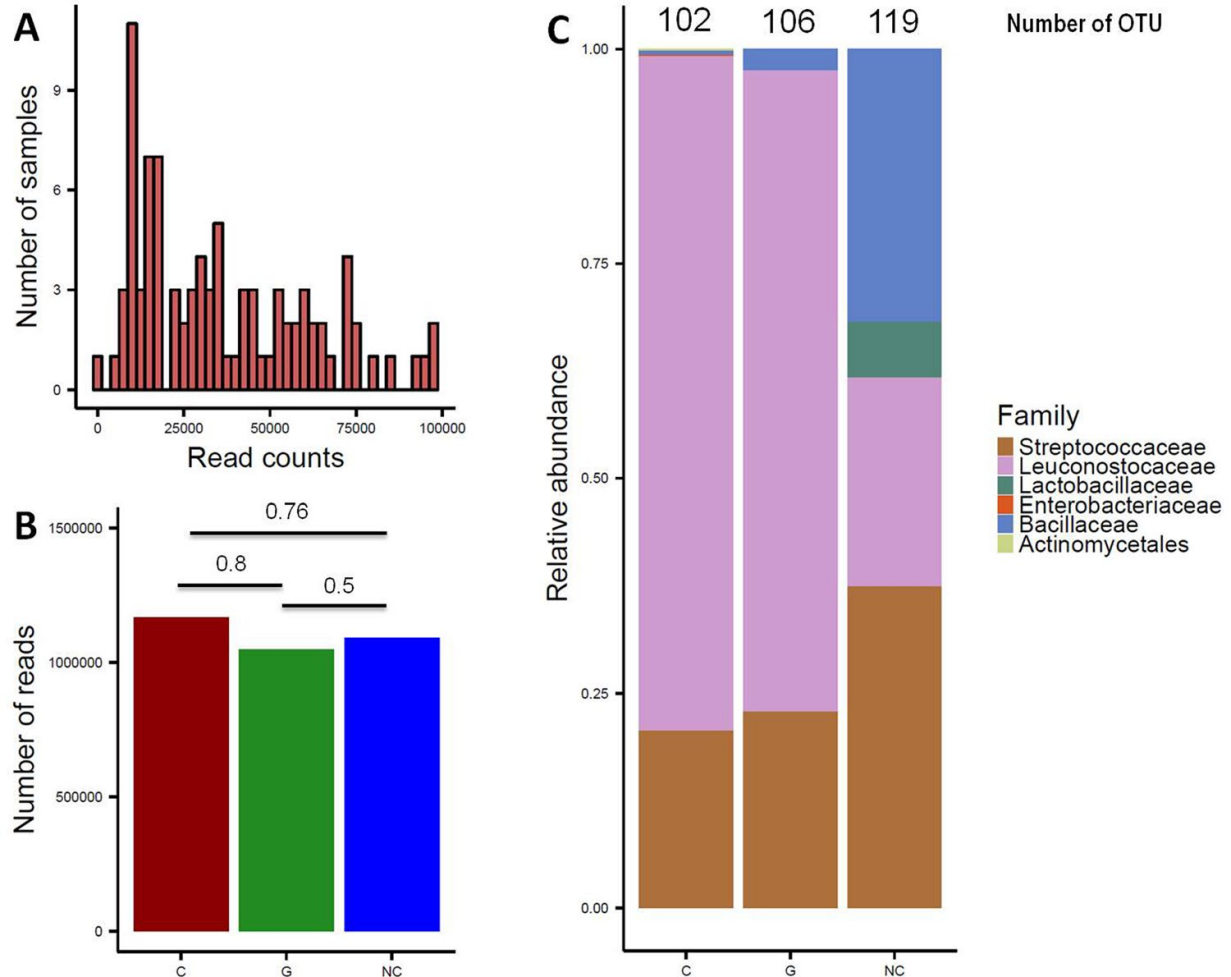
Description of data generated by sequencing and mothur analysis. (**A**) Sequencing depth in all samples concatenated. (**B**) Sequencing depth according to larval status. (**C**) Relative abundance of bacterial families across the three groups (C: cancerous; G: non-cancerous; NC: control). For the sake of clarity, families with a relative abundance below 2% are not included.

After having applied all filtering methods described in the previous section, our dataset consisted of 897 unique nearly full-length high-quality clones (*i.e.*, groups of identical sequences) representing 90 larva samples (which we refer to as libraries). This dataset excluded 45 536 clones that had a length inferior to 90 bp (we expected sequences with an average length of 180 bp) and that were not correctly aligned to reference sequences (differed from 95% of the sequences). In addition, 44 421 clones were eliminated because they presented one base of difference with the reference sequences or because they were identified as chimeric by Mothur software. Finally, 1040 clones were removed because they were represented by one sequence only. From the 897 clones, clustering with Mothur, using an average neighbor algorithm with 97% of similarity with the reference database, has created 179 distinct OTUs and corresponded to more than three billion sequences (*i.e.*, reads). The libraries varied in terms of sequencing depth, ranging from 10^2^ to 10^5^ reads per sample (Fig. 2A). However, sequencing depth was comparable between the three groups of larvae (p-values for each combination from the Mann-Whitney test: C-G = 0.8, NC-G = 0.5, NC-C = 0.76; Fig. 2B). Finally, controls for dissection, extractions, and PCR did not show bacterial contamination, and taxa of interest were absent from microbiota-free larvae (see Figs S1, S2 in supplementary materials).

The bacterial community of our libraries essentially comprised four phyla: Actinobacteria, Bacteroidetes, Proteobacteria, and Firmicutes; this latter was by far the most prevalent (99% of total OTUs). Even though the number of OTUs was high, most of families were poorly represented (low number of sequences, very few samples). Four bacterial families of Firmicutes accounted for nearly all the diversity of our libraries (98% of total OTUs) (Fig. 2C). These included Leuconostococaceae, which was the predominant phylotype (60% of Firmicutes), Streptococaceae (30%), Bacillaceae (12%), and Lactobacillaceae (2%). Microbial compositions were highly conserved between individuals at the phylum and family levels (Fig. S3 in supplementary materials). However, microdiversity (*i.e.*, diversity in rare OTUs) was highly variable at the inter-individual level. In fact, when we considered families with a relative abundance superior to 0.02% (assuming this avoids observing random sequencing errors), we found that the number of OTUs ranged from 35 to 5 by sample, with an average of 19 OTUs per sample.

**Figure 3:**
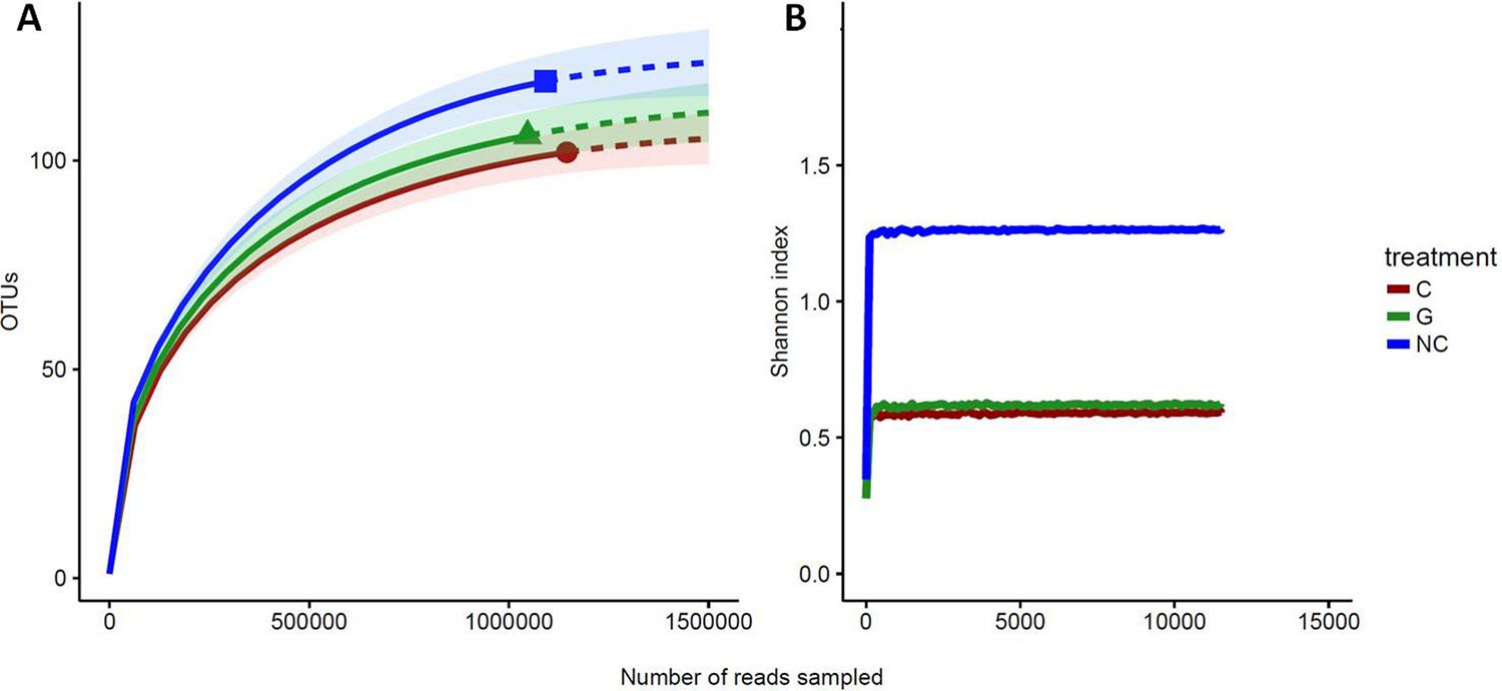
Rarefaction analysis of 16S rRNA gene clone libraries. (**A**) Observed rarefaction curves generated with the iNEXT package. Solid lines represent the observed accumulation with the number of reads sampled, and dashed lines represent the extrapolated accumulation considering 25% more reads. Shaded areas are the 95% confidence intervals. (**B**) Shannon’s index rarefaction curves (C: cancerous; G: non-cancerous; NC: control).

Rarefaction analyses were used to determine if the sampling effort was sufficient to correctly describe the bacterial community. It showed that larval groups differed slightly in richness and were sampled at comparable depths (Fig. 3). Even if the rarefaction curves based on the number of OTUs did not reach a plateau (and therefore convergence), extrapolations with 25% supplementary reads showed that the sequencing depth for each group was satisfactory (Fig. 3A). Moreover, the Shannon diversity estimates were stable at the observed sequencing depth and even with lower one (Fig. 3B). This suggests that although new phylotypes could be expected with additional sequencing, most of the diversity was already captured. Finally, Good’s estimator of coverage for the total library was greater than 99%.

### Characterization of parental effects on intestinal microbiota

**Figure 4:**
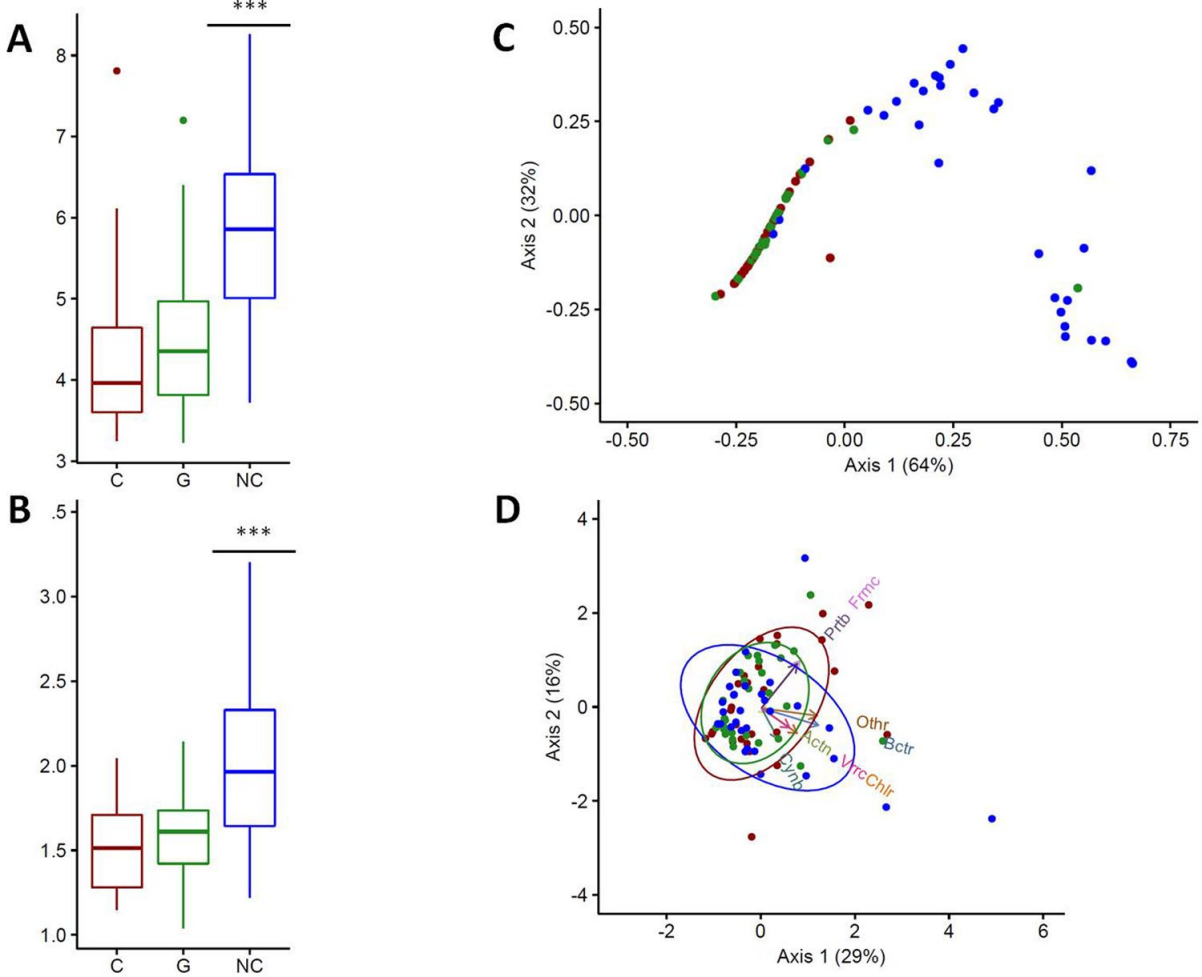
Genetic background affects the diversity and structure of the bacterial communities of larvae. (**A**) Alpha diversity and (**B**) Simpson’s index of evenness for each group of larvae (C: cancerous; G: non-cancerous; NC: control). (**C**) Principal coordinates analysis and (**D**) principal component analysis for each group of larvae. Blue dots represent control individuals, green dots non-cancerous individuals, and red dots cancerous individuals. In (**D**), arrows show the contribution of each principal phylum to the dimensions (Actn: Actinobacteria, Bctr: Bacteroidetes, Chlr: Chloroflexi, Cynb: Cyanobacteria, Frmc: Firmicute, Prtb: Proteobacteria, Vrrc: Verrumicrobia). Ellipses consider normal data. ***p-value < 0.0001.

All diversity estimators indicated that control larvae had a more diverse bacterial community than cancerous and non-cancerous larvae coming from cross 2 (descendants carrying oncogenic mutations) (Table 1). In fact, control larvae tended to have a higher number of OTUs and a higher Shannon’s diversity index value than the other two groups, whatever the cancerous status. In addition, this difference was found to be significant with respect to the alpha diversity (W = 287, p-value < 0.0001) and Simpson index of evenness (W = 394, p-value < 0.0001) (Fig. 4A and B).

**Table 1:** Drosophila microbial flora diversity estimates according to groups of interest.

Groups	No. of samples	No. of clones	No. of OTUs (phylum level)	No. of OTUs (family level)	Observed alpha diversity	Shannon’s index of diversity	Simpson’s index of diversity	Simpson’s eveness
Cancerous (C)	30	1145021	19	102	4.3	0.59	1.577	1.52
Non cancerous (G)	30	1046822	16	106	4.5	0.62	1.569	1.58
Control (NC)	30	1092023	21	119	5.9	1.26	3.25	2.08
Total	90	3283855	28	179	4.9	0.64	1.7	1.73

Multivariate statistical analyses were performed to compare the overall structure of the intestinal microbiota for all samples. PCoA based on the relative abundance of OTUs revealed a separation between control individuals and the two other groups according to the first two principal component scores, which accounted for 64% and 32% of the total variation respectively (96% in total; Fig. 4C). However, PCoA also showed that the bacterial microbiome of cancerous larvae was similar to that of non-cancerous larvae. Finally, PCA also revealed a difference in the structure of microbial communities. We observed that the differentiation between larvae from distinct cross was driven by different phyla (Fig. 4D). Thus, rarer phyla such as Verrumicrobia and Cyanobacteria were associated with control larvae, whereas dominant phyla (*e.g.*, Firmicutes and Proteobacteria) were correlated with cancerous and non-cancerous groups.

### Influence of cancerous status on bacterial families residing in larval intestines

**Figure 5:**
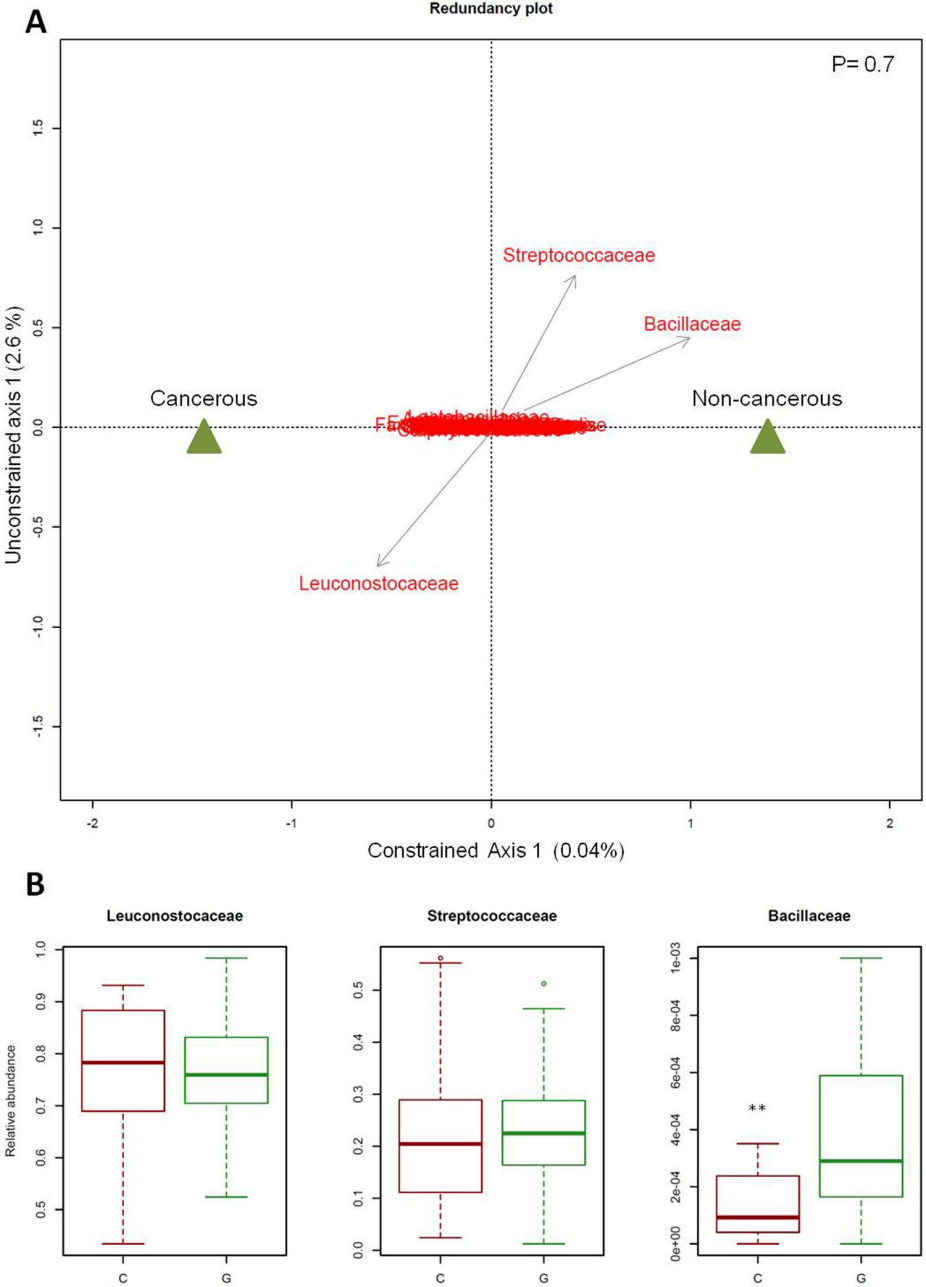
Influence of cancerous status on bacterial microbiota. (**A**) Biplot of the redundancy analysis considering constrained ordination model based on the relative abundance of operational taxonomic units (OTUs) (97% similarity level). OTUs with a relative abundance superior to 0.02% are indicated in red. The triangles show the centroids for factor constraints. The p-value of the Monte Carlo Permutation Test is shown in the upper right. (**B**) Relative abundance of three families of interest in the cancerous (C) and non-cancerous (G) groups. **p-value < 0.001.

Despite the relatively tight clustering of cancerous and non-cancerous samples, RDA analysis allowed the identification of small differences in microbiota composition. In fact, the two first axes explained only 0.04% (constrained axis) and 2.6% (unconstrained axis) of the total variation respectively and a Monte Carlo Permutation Test showed that the constrained ordination model produced by the RDA was not significant (p-value = 0.7). Thus, the variations observed in the relative abundances of OTUs cannot be explained by cancerous status. In other words, the presence of a tumor was not associated with a modification in the microbiota structure, in agreement with the results from previous multivariate analyses. However, we identified three OTUs as key variables which had at least 3% of the variability in their values explained by the constrained axis and at least 40% by the unconstrained axis (Fig. 5A). On the RDA biplot, we observed that Bacillaceae and Streptococcaceae were more abundant in the microbiota of non-cancerous larvae contrary to Leuconostocaceae, which were more common in cancerous larvae. Mann-Whitney tests showed that only the relative abundance of Bacillaceae was significantly different between cancerous and non-cancerous groups (W = 211, p-value = 0.0004; corresponding to a risk α = 0.055 for 136 OTUs, according to Šidák correction). It is also worth noting that family Bacillaceae was essentially made up of the Bacillus genus.

## Discussion

The aim of our study was to explore if non-digestive cancers, especially brain cancer, could be associated with a modification in the composition of intestinal microbiota. We characterized the diversity and structure of the intestinal microbiota in Drosophila larvae developing a cancer of the eye-antennal disc. First, we observed that cancerous and non-cancerous individuals with oncogenic mutations were not distinct according to the various diversity estimators that we employed, *i.e.*, the composition of individuals’ bacterial communities were similar. However, this composition was significantly different from control larvae, which did not carry the oncogenic mutations. Second, we identified a bacterial signature of the cancerous status at the family level: cancerous larvae had a significantly lower relative abundance of Bacillaceae than individuals who did not develop the tumor. Thus, for the first time, we showed that brain cancer could be associated with alterations in the intestinal microbial community.

Two hypotheses may explain the observed parental effect (*i.e*., parents play a role in determining offspring’s phenotype through non-genetic or genetic influences) on the microbiota structure and diversity of larvae groups. First, it has been shown that larvae acquire the bacteria that compose their microbiota by feeding on adult feces^39^. Females were genetically identical and harvested under identical conditions in the two crosses and thus cannot affect microbiota of descendants distinctly. However, as males came from two distinct lines, they could show divergent microbiota after extended husbandry isolation (as suggested in mice^40^). Thus, we hypothesize that the parental effect observed in our study may result from transmission of bacterial species from different males. Nevertheless, it has been shown that several laboratory lines of Drosophila have a low microbial diversity if they have been fed with the same fly media^41^. A second hypothesis relies on the recent observation of an association between host genome and microbiome^42^. In this context, oncogenic mutations could thus have an additional and significant effect on bacterial community structure. This hypothesis is particularly interesting because it implies that mutations, through modifying an individual’s phenotype, may alter intestinal microbiota even before cancer emerges. As scrib is involved in cell migration and polarity^43^, mutations could significantly affect the integrity of the gut epithelium, which is known to affect associated bacteria species^44^. The characterization of microbiota structure in larvae harboring other mutations triggering brain cancer (see^45^ for examples) could provide crucial information to disentangle the effect of genetic mutations on microbiota. Further studies should also determine the part of microbiota structure that could be attributed to oncogenic mutations or to parental effects. For example, it would be necessary to reproduce our experiment by separating males and females before egg-laying. Another step would be to compare the microbiota of parent flies to those of their descendants. Finally, the observation of naturally-acquired microbiota in axenic larvae, bearing oncogenic mutation or not, may be also relevant to report in which measure mutations impact the colonization by bacterial species. Such experiments are definitely crucial before being able extrapolating to human diseases because intestinal microbiota is not considered to be heritable—or to a very low extent^46^—whereas several oncogenic mutations could be^47^.

The bacterial signature between cancerous and non-cancerous individuals is due to the Bacillaceae family. However, our study does not test if a higher relative abundance of this family protects against cancer development or, conversely, if the development of cancer cells affects Bacillaceae populations specifically. It has recently been suggested that the efficiency of the immune system may depend on the immunogenicity of microbiome lipopolysaccharides (LPS)^48^. In this study, authors demonstrated that *Escherichia coli* is highly immunogenic because of LPS at its surface and that early-life infection by this bacteria may contribute to a more equilibrated immune system. Bacillaceae is one of the rare Firmicutes families that share a specific LPS with *E. coli* ^49^, suggesting that they could also share the same immunogenicity. This expectation is supported by the potential of *Bacillus* spp.*—*the dominant genus of the family in our study—to increase mortality in other insect species^50^, which could reflect an over-response of the immune system. Even though we did not observe such mortality, which could be the result of co-evolution, we can expect that the immune system is still stimulated by the presence of the bacteria, at least at a low level. Thus, we hypothesize that species of Bacillaceae could help to eliminate cancer cells at the beginning of carcinogenesis, and therefore avoiding cancer emergence for individuals carrying oncogenic mutations. To confirm the impact of Bacillaceae species on cancer development and the accuracy of our hypothesis, additional experiments are clearly necessary. First, the comparison of axenic, microbiota-colonized and mono-associated axenic larvae (with specific colonization by Bacillaceae species) should allow a better characterization of immune response pathways triggered by these bacteria, as well as the intrinsic impact of this family on cancer cell accumulation. If the role of Bacillaceae family is confirmed, further studies should investigate its mechanistic effects by exploring the effect of an injection of specific LPS on cancer cell accumulation. Alternatively, colonization of axenic larvae with modified Bacillaceae, unable to produce LPS, could help distinguishing if LPS production is the only active mechanism involved in cancer development.

Drosophila is a recognized model for cancer study^45^ and may be useful in the study of questions that may not be addressable with human biological data. For example, human bacterial diversity is usually measured from fecal samples, but the degree to which the composition and function of fecal samples differ from mucosal samples remains unclear^51^. With animal models, this constraint is removed, and species richness can be examined throughout the whole intestine. One important limitation for extrapolating our results to humans relies in their dramatically different microbiota composition compared to Drosophila. Most human studies linking microbiota structure and diversity to cancer (principally colorectal cancer) have identified changes in the proportions of Bacteroides in communities (see^52^ for a review). However, Bacteroides are almost absent from the microbiota of Drosophila larvae, which is largely dominated by Firmicutes. In addition, characterization of microbiota composition at species level has not been considered in our study because of the small length of our 16s fragment as well as our sample size. Further studies, exploring bacterial signature at species level through the sequencing of full-length 16s RNA^53^, may allow identifying specific taxa implied in carcinogenesis. In this context, the long-distance crosstalk between brain cancer and intestinal microbiota should be investigated in humans in order to identify precise bacterial signatures of brain cancer. Another caveat of our study is that we characterized microbiota structure and diversity at the 3^rd^ instar larval stage, which is reached after only a few days of development. Thus, this model does not include long-term processes that could occur in long-living animals. A first step could be to reproduce our study in an adult insect model where carcinogenesis may be more progressive (see, *e.g.*,^54^). Finally, alimentation is a key factor to take into account when investigating microbiota modification. Alimentation often differs between individuals in nature and deeply affects microbiota (both in humans^55^ and insects^41^). In addition, it has been proposed that microbiota may influence nutrition^56^ and/or immunity, both of which can affect cancer risk^57,58^. Since we always used the same food media for all individuals, we may underestimate the difference that could exist between cancerous and non-cancerous individuals in natural situations (especially if cancerous individuals experience a different nutrition). Further studies should consider interactions of alimentation with the gut–brain axis to accurately identify bacterial cancer signatures.

The three-way interconnection between intestinal microbiome, cancer, and the immune system is increasingly recognized^16^. Even though our study gives preliminary results, it shows for the first time the possibility of long-distance effects of intestinal microbiota on cancer in distant organs. One important task that remains is to identify the orientation and mechanisms of the long-distant communication between brain and gut. In fact, alterations in microbiota communities could be neutral, detrimental, or beneficial to cancer, and studies should be undertaken to identify the causality of these changes. Such studies could allow the identification of species associated with a decreased risk of cancer that could be used as a preventive measure (*e.g*., administration of probiotics), and drugs could also be designed to mimic their influence on the immune system to improve cancer cell elimination. Finally, increasing our knowledge about pro-tumoral species could also have important applications, for example, they could serve as biomarkers for early detection of cancer risk, or they could be targeted by therapeutic strategies such as narrow-spectrum antibiotics.

## Electronic supplementary material

Supplementary Information

## References

[1] Graf D (2015). Contribution of diet to the composition of the human gut microbiota. Microb. Ecol. Health Dis..

[2] David LA (2014). Host lifestyle affects human microbiota on daily timescales. Genome Biol..

[3] Turnbaugh PJ (2006). An obesity-associated gut microbiome with increased capacity for energy harvest. Nature.

[4] Kau AL, Ahern PP, Griffin NW, Goodman AL, Gordon JI (2011). Human nutrition, the gut microbiome and the immune system. Nature.

[5] Hanahan D, Weinberg RA (2011). Review Hallmarks of Cancer: The Next Generation. Cell.

[6] Peto J (2001). Cancer epidemiology in the last century and the next decade. Nature.

[7] Thomas RM, Jobin C (2015). The Microbiome and Cancer: Is the ‘Oncobiome’ Mirage Real?. Trends in Cancer.

[8] Schwabe, R. F. & Jobin, C. The microbiome and cancer. *Nat. Rev. Cancer* **13**, 800–12 (2013). 10.1038/nrc3610 PMC3986062 24132111

[9] Sartor, R. B. & Mazmanian, S. K. Intestinal Microbes in Inflammatory Bowel Diseases. *Am. J. Gastroenterol. Suppl*. **1**, 15–21 (2012).

[10] Jangi, S. *et al*. Alterations of the human gut microbiome in multiple sclerosis. *Nat. Commun.* **7**, 12015 (2016). 10.1038/ncomms12015 PMC4931233 27352007

[11] Francescone, R., Hou, V. & Grivennikov, S. I. Microbiome, inflammation, and cancer. *Cancer J.*, **20**, 181–9 (2014). 10.1097/PPO.0000000000000048 PMC4112188 24855005

[12] Grivennikov SI (2012). Adenoma-linked barrier defects and microbial products drive IL-23/IL-17-mediated tumour growth. Nature.

[13] Bhattacharya N (2016). Normalizing Microbiota-Induced Retinoic Acid Deficiency Stimulates Protective CD8+T Cell-Mediated Immunity in Colorectal Cancer. Immunity.

[14] Belkaid Y, Hand TW (2014). Role of the Microbiota in Immunity and Inflammation. Cell.

[15] Maynard CL, Elson CO, Hatton RD, Weaver CT (2012). Reciprocal interactions of the intestinal microbiota and immune system. Nature.

[16] Zitvogel L, Ayyoub M, Routy B, Kroemer G (2016). Microbiome and Anticancer Immunosurveillance. Cell.

[17] Dapito Dh (2013). Promotion of Hepatocellular Carcinoma by the instestinal Microbiota and TLR4. Cancer Cell.

[18] Xuan C (2014). Microbial Dysbiosis Is Associated with Human Breast Cancer. PLoS One.

[19] Schroeder BO, Bäckhed F (2016). Signals from the gut microbiota to distant organs in physiology and disease. Nat. Med..

[20] Collins SM, Surette M, Bercik P (2012). The interplay between the intestinal microbiota and the brain. Nat. Rev. Microbiol..

[21] Mayer EA (2011). Gut feelings: the emerging biology of gut-brain communication. Nat. Rev. Neurosci..

[22] Aidy SE, Dinan TG, Cryan JF (2014). Immune modulation of the brain-gut-microbe axis. Front. Microbiol..

[23] Pagliarini RA, Xu T (2003). A Genetic Screen in Drosophila for Metatstatic Behavior. Science (80-.)..

[24] Salter SJ (2014). Reagent and laboratory contamination can critically impact sequence-based microbiome analyses. BMC Biol..

[25] Milani, C. *et al*. Assessing the Fecal Microbiota: An Optimized Ion Torrent 16S rRNA Gene-Based Analysis Protocol. *PLoS One* **8** (2013). 10.1371/journal.pone.0068739 PMC3711900 23869230

[26] Schloss PD (2009). Introducing mothur: open-source, platform-independent, community-supported software for describing and comparing microbial communities. Appl. Environ. Microbiol..

[27] Schloss PD (2010). The Effects of Alignment Quality, Distance Calculation Method, Sequence Filtering, and Region on the Analysis of 16S rRNA Gene-Based Studies. PLoS Comput. Biol..

[28] Quast C (2013). The SILVA ribosomal RNA gene database project: improved data processing and web-based tools. Nucleic Acids Res..

[29] Wang Q, Garrity GM, Tiedje JM, Cole JR (2007). Naive Bayesian classifier for rapid assignment of rRNA sequences into the new bacterial taxonomy. Appl. Environ. Microbiol..

[30] Konstantinidis, K. T. & Tiedje, J. M. Genomic insights that advance the species definition for prokaryotes.at https://www.ncbi.nlm.nih.gov/pmc/articles/PMC549018/pdf/pnas-0409727102.pdf (2004). 10.1073/pnas.0409727102 PMC549018 15701695

[31] McMurdie, P. J. & Holmes, S. Phyloseq: An R Package for Reproducible Interactive Analysis and Graphics of Microbiome Census Data. *PLoS One* **8** (2013). 10.1371/journal.pone.0061217 PMC3632530 23630581

[32] Staley JT (2006). The bacterial species dilemma and the genomic-phylogenetic species concept. Philos. Trans. R. Soc. Lond. B. Biol. Sci..

[33] Jovel J (2016). Characterization of the gut microbiome using 16S or shotgun metagenomics. Front. Microbiol..

[34] Hsieh, T. C., Ma, K. H. & Chao, A. iNEXT: An R package for rarefaction and extrapolation of species diversity (Hill numbers). *Methods Ecol. Evol*. 1451–1456, doi:10.1111/2041-210X.12613 (2016).

[35] Lau M (2014). Functions for re-sampling a community matrix to compute diversity indices at different sampling levels. R Packag. version.

[36] Good IJ (1953). The Population Frequencies of Species and the Estimation of Population Parameters. Biometrika.

[37] Šidák Z (1967). Rectangular Confidence Regions for the Means of Multivariate Normal Distributions. J. Am. Stat. Assoc..

[38] R Development Core Team. R: A Language and Environment for Statistical Computing (2011).

[39] Erkosar B, Leulier F (2014). Transient adult microbiota, gut homeostasis and longevity: Novel insights from the Drosophila model. FEBS Lett..

[40] Ubeda C (2012). Familial transmission rather than defective innate immunity shapes the distinct intestinal microbiota of TLR-deficient mice. J. Exp. Med..

[41] Chandler, J. A., Lang, J., Bhatnagar, S., Eisen, J. A. & Kopp, A. Bacterial communities of diverse Drosophila species: Ecological context of a host-microbe model system. *PLoS Genet*. **7** (2011). 10.1371/journal.pgen.1002272 PMC3178584 21966276

[42] Abdul-Aziz MA, Cooper A, Weyrich LS (2016). Exploring relationships between host genome and microbiome: New insights from genome-wide association studies. Front. Microbiol..

[43] Nola S (2008). Scrib regulates PAK activity during the cell migration process. Hum. Mol. Genet..

[44] Peterson LW, Artis D (2014). Intestinal epithelial cells: regulators of barrier function and immune homeostasis. Nat. Rev. Immunol..

[45] Gonzalez C (2013). Drosophila melanogaster: a model and a tool to investigate malignancy and identify new therapeutics. Nat. Rev. Cancer.

[46] Yatsunenko T (2012). Human gut microbiome viewed across age and geography. Nature.

[47] Futreal PA (2004). A census of human cancer genes. Nat. Rev. Cancer.

[48] Vatanen T (2016). Variation in Microbiome LPS Immunogenicity Contributes to Autoimmunity in Humans. Cell.

[49] Vollmer W (2011). Bacterial outer membrane evolution via sporulation?. Nat. Chem. Biol..

[50] Tsagou V, Lianou A, Lazarakis D, Emmanouel N, Aggelis G (2004). Newly isolated bacterial strains belonging to Bacillaceae (Bacillus sp.) and Micrococcaceae accelerate death of the honey bee mite, Varroa destructor (V. jacobsoni), in laboratory assays. Biotechnol. Lett..

[51] Eckburg PB (2010). Diversity of the Human Intestinal Microbial Flora Paul. Science (80-.)..

[52] Sun J, Kato I (2016). Gut microbiota, inflammation and colorectal cancer. Genes Dis..

[53] Shin, J. *et al*. Analysis of the mouse gut microbiome using full-length 16S rRNA amplicon sequencing. Nat. Publ. Gr., doi:10.1038/srep29681 (2016). 10.1038/srep29681 PMC4944186 27411898

[54] Martorell Ò (2014). Conserved mechanisms of tumorigenesis in the Drosophila adult midgut. PLoS One.

[55] Hamilton MK, Boudry G, Lemay DG, Raybould HE (2015). Changes in intestinal barrier function and gut microbiota in high-fat diet-fed rats are dynamic and region dependent. Am J Physiol Gastrointest Liver Physiol.

[56] Alcock, J., Maley, C. C. & Aktipis, C. A. Is eating behavior manipulated by the gastrointestinal microbiota? Evolutionary pressures and potential mechanisms. *Bioessays* 940–949, doi:10.1002/bies.201400071 (2014). 10.1002/bies.201400071 PMC4270213 25103109

[57] Loo, T. M. *et al*. Gut Microbiota Promotes Obesity-Associated Liver Cancer through PGE2-Mediated Suppression of Antitumor Immunity. *Cancer Discov*. CD-16–0932, doi:10.1158/2159-8290.CD-16-0932 (2017). 10.1158/2159-8290.CD-16-0932 28202625

[58] Kau AL, Ahern PP, Griffin NW, Goodman AL, Jeffrey I (2012). Human nutrition, the gut microbiome, and immune system: envisioning the future. Nature.

