# Supplementary material for: Can intestinal microbiota be associated with non-intestinal cancers?
Source: Sci Rep. 2017 Oct 5;7:12722. doi: 10.1038/s41598-017-11644-9 (PMC5629204; doi:10.1038/s41598-017-11644-9)
Supplement: Supplementary file 1 — Supplementary Information [file 41598_2017_11644_MOESM1_ESM.doc]

**Can intestinal microbiota be associated with non-intestinal cancers? -** **Supplementary materials**

Camille Jacqueline , Lionel Brazier, Dominique Faugère, François Renaud, Frédéric Thomas, Benjamin Roche

1. *Primers*

***Table 1: Primers used in this study (****Milani et al. 2013).*

| **Primer name** | **Adapter sequence** | **Key** | **Tag barcode** | **GAT** | **Primer sequence**  **(5'-3')** |
| --- | --- | --- | --- | --- | --- |
| Probio_Uni | CCATCTCATCCCTGCGTGTCTCCGAC | TCAG | TTGGAGTGTC | GAT | CCTACGGGRSGCAGCAG |
| Probio_Rev | CCTCTCTATGGGCAGTCGGTGAT |  |  |  | ATTACCGCGGCTGCT |

1. *Controls*

The profiles of all controls (dissection, extraction, and PCR) analyzed with a 2100 Bioanalyser show no detectable amplification, supporting the absence of bacterial contamination during the manipulation (Fig. S1). Nevertheless, we conducted the sequencing for one control extraction and for microbiota-free larvae. The control extraction did not have the common family found in our samples, but rather had non-typical bacterial families of the Drosophila gut (Fig. S2). Microbiota-free larvae had high relative abundances of Leuconostocaceae and Streptococaceae, which are also the most abundant families in samples. Thus, these families could be present in the general cavity of larva bodies. However, taxa of interest, such as Lactobacillaceae and Bacillaceae, were relatively rare in these samples and thus specific to intestinal microbiota.

*
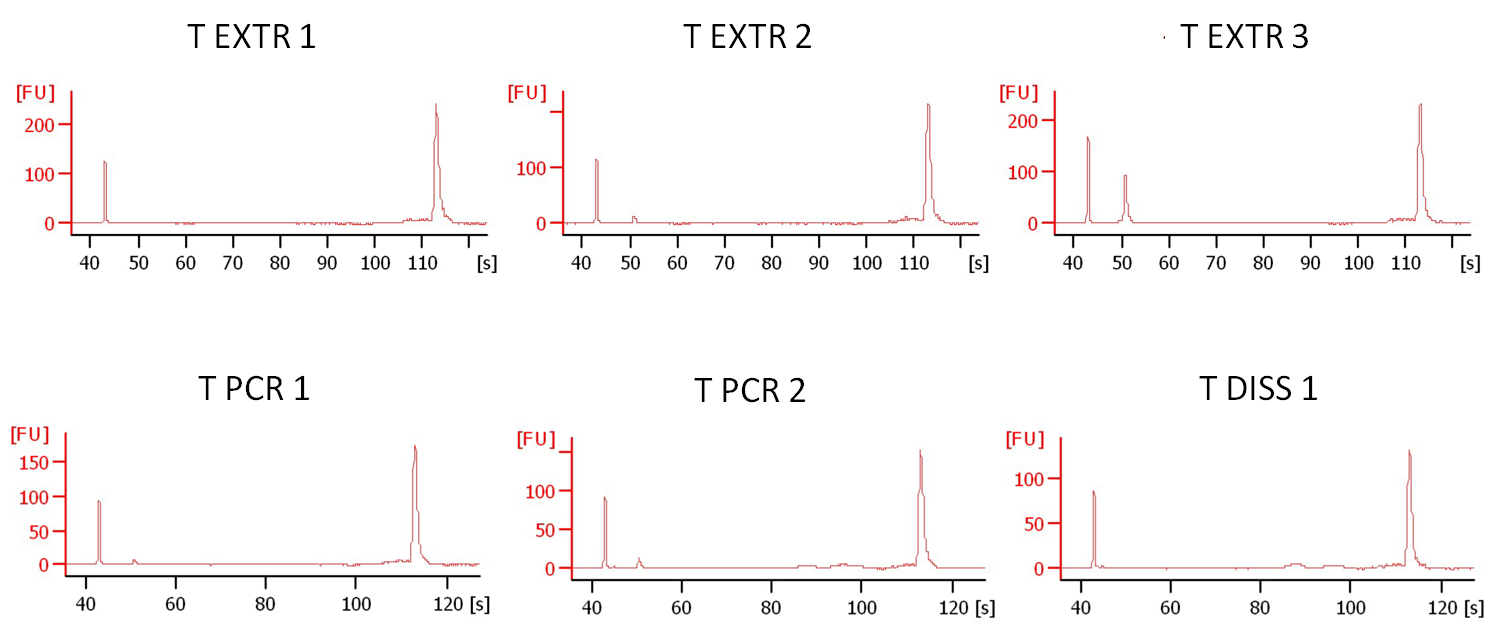
*

Figure S1. **Profiles of fluorescence obtained with the Bioanalyser.** The absence of a peak around 80s shows that bacterial DNA were not amplified.


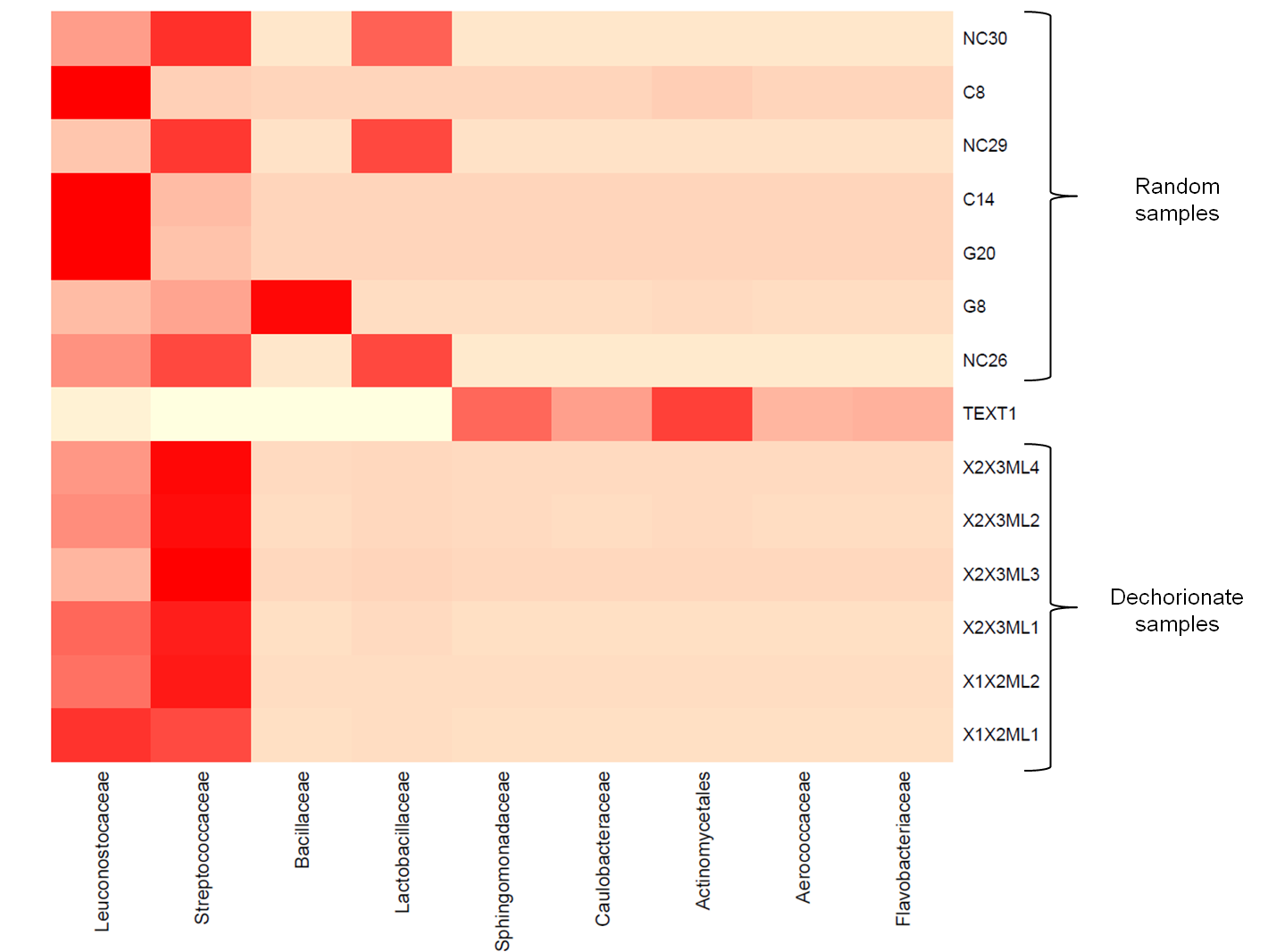


**Figure S2. Heat map showing the relative abundances of the main bacterial families in the guts of microbiota-free samples compared to randomly selected samples.**

1. *Consistence in diversity across samples.*

**
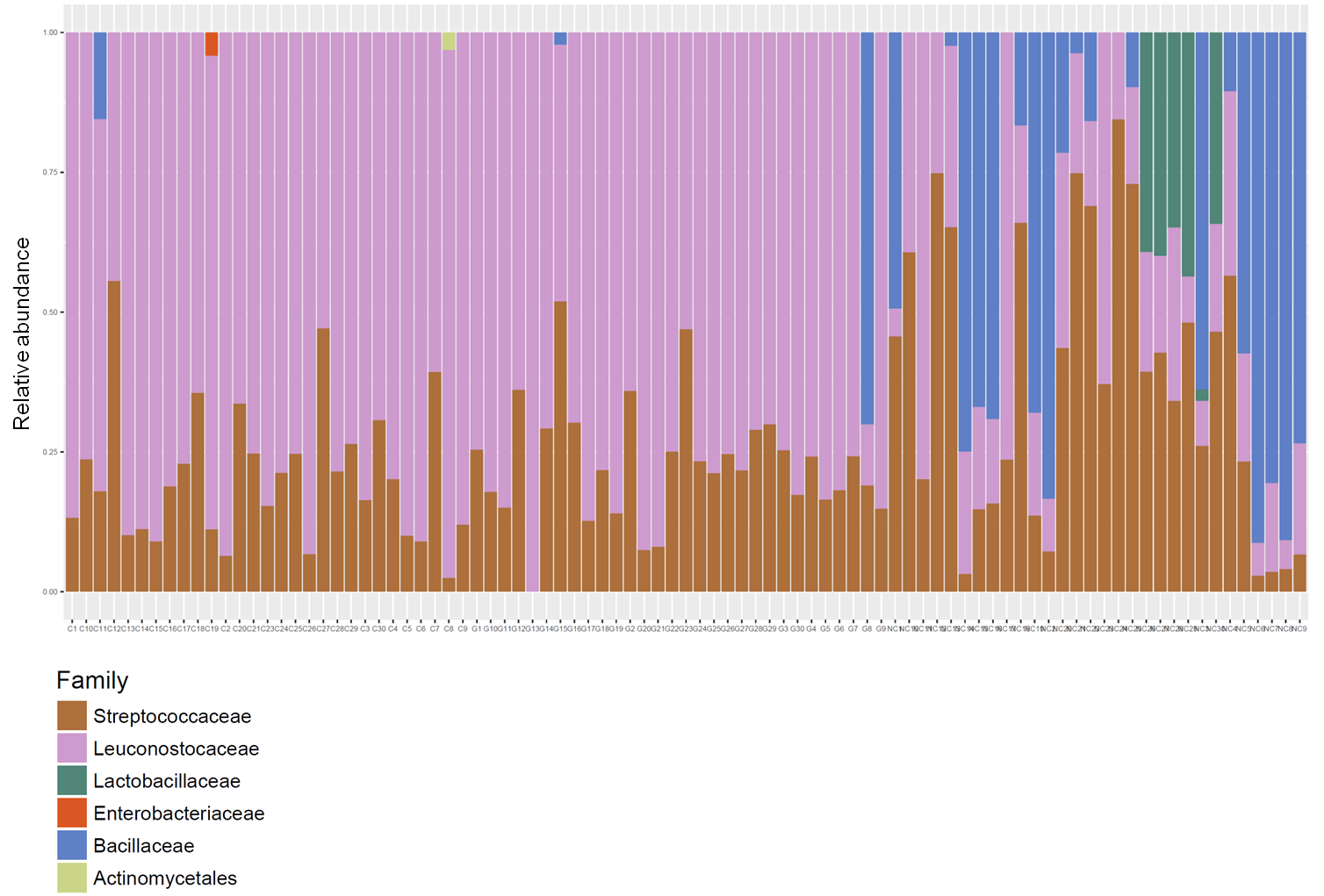
**

**Figure S3. Relative abundance at the family level across samples.** Only families with a relative abundance greater than 2% are included here.
